# Service Providers' Perspectives on Family‐Centred Practice Frameworks in Paediatric Disability Services: A Scoping Review

**DOI:** 10.1111/cch.70376

**Published:** 2026-11-08

**Authors:** Yanlin Liu, Agnes Shiel, Hazel Killeen

**Affiliations:** ^1^ Discipline of Occupational Therapy, School of Health Sciences University of Galway Galway Ireland

## Abstract

**Background:**

Family‐centred practice (FCP) is a core principle of paediatric disability and rehabilitation services. Although many frameworks, models, programmes and tools have been developed to support FCP, translating these approaches into routine practice remains complex. Service providers play a key role in implementation, yet evidence on how they perceive, use and experience FCP frameworks across paediatric disability‐related services remains fragmented. This scoping review mapped the literature on service providers' perspectives of FCP frameworks for children with disabilities and their families.

**Methods:**

A scoping review was conducted in accordance with established guidance and reported following the PRISMA Extension for Scoping Reviews checklist. Searches were conducted in CINAHL Complete, APA PsycINFO, SocINDEX with Full Text, Academic Search Complete, Scopus and MEDLINE via PubMed. Full‐text empirical journal articles were included if they reported service providers' perspectives on FCP frameworks in paediatric disability‐related contexts. Data were charted and synthesised descriptively and narratively.

**Results:**

Twenty‐one studies were included. The studies examined conceptual and guiding frameworks, service delivery models, coaching and relational approaches, intervention and training models and assessment and collaborative planning tools. Six themes were identified: professional role transformation, relational and communication competencies, collaborative goal setting, barriers to implementation, training and professional support needs and the influence of context and service delivery. Service providers generally valued FCP frameworks for supporting collaboration and meaningful goal setting. Implementation was constrained by time pressure, workload, tensions with expert‐led practice, limited training, resource demands and contextual fit.

**Conclusion:**

FCP frameworks can support service providers to operationalise family‐centred principles, but in isolation, they are not sufficient to ensure practice change. Implementation requires professional role development, relational competencies, training, supervision, organisational support and service conditions that enable sustained collaboration with families.

## Summary

- Family‐centred practice frameworks can support collaboration, goal setting and shared planning in paediatric disability services.
- Service providers' perspectives show that implementation requires shifts in professional roles and therapeutic relationships.
- Relational and communication competencies are central to family‐centred practice in everyday service delivery.
- Time pressure, workload, limited training and resource constraints restrict implementation.
- Future implementation should include professional development, supervision, organisational support and contextual adaptation.

## 1 Introduction

Family‐centred practice (FCP) is a central principle in paediatric rehabilitation, early intervention and children's disability services, where children's participation and well‐being are understood in relation to family routines, relationships, environments and everyday life contexts (Dunst and Espe‐Sherwindt 2016; King et al. 2017; McCarthy and Guerin 2022). In practice, FCP involves respectful partnerships, shared decision‐making, information sharing and recognition of parents and caregivers as experts in their child's daily life (McCarthy and Guerin 2022).

Despite strong conceptual and policy support, translating FCP from principle into routine practice remains challenging in paediatric disability‐related services (Lage et al. 2024; McCarthy and Guerin 2022). FCP requires service providers to move beyond expert‐led or impairment‐focused intervention and work in ways that support family priorities, shared expertise and collaborative decision‐making (D'Arrigo et al. 2020; Dunst and Espe‐Sherwindt 2016; Lage et al. 2024). This shift can place considerable demands on service providers because family‐centred work requires relational competence, coaching skills, emotional responsiveness and the ability to balance professional expertise with family knowledge (D'Arrigo et al. 2020; Harniess et al. 2022; Lage et al. 2024). Implementation is also influenced by workforce capacity, leadership, training, resources and the local service context (Damschroder et al. 2022; McCarthy and Guerin 2022; Nilsen 2015).

A range of frameworks, models, programmes, structured approaches and tools has been developed to translate FCP principles into service delivery and professional practice (King et al. 2017; McCarthy and Guerin 2022). Terminology varies across the literature, and no universally accepted practice model for operationalising FCP has been established (Kokorelias et al. 2019; McCarthy et al. 2021). In this review, “FCP frameworks” is used as an umbrella term for identifiable and structured frameworks, models, programmes, approaches or tools designed to translate family‐centred principles into practice. These approaches contain principles, components, procedures or processes that guide service planning, delivery or implementation and involve service providers in their application. This definition distinguishes an actionable approach from FCP considered only as a general philosophy or concept.

Previous reviews have examined family‐centred care processes, family‐oriented paediatric rehabilitation and parent engagement (Harniess et al. 2022; King et al. 2017; McCarthy and Guerin 2022). However, evidence on service providers' experiences of using the different approaches intended to support FCP has not been mapped across paediatric disability‐related services. This perspective is of critical importance as service providers are tasked with interpreting these approaches, introducing them to families and negotiating their use within existing roles, routines and service parameters (D'Arrigo et al. 2020; Damschroder et al. 2022; Lage et al. 2024). Mapping this evidence may inform service providers, service managers and implementation leaders when selecting, applying and supporting structured approaches to FCP in paediatric disability‐related services.

This scoping review therefore aimed to map service providers' perspectives and experiences of applying and implementing FCP frameworks in paediatric disability‐related services, including perceived benefits, limitations, barriers, facilitators and support needs. The frameworks were also identified and described to provide context for interpreting these perspectives. The review question was: What are service providers' perspectives on FCP frameworks for children with disabilities and their families?

## 2 Methods

### 2.1 Study Design

The review was informed by established scoping review methodology and JBI guidance (Arksey and O'Malley 2005; Levac et al. 2010; Peters et al. 2022) and reported following the PRISMA‐ScR checklist (Tricco et al. 2018). No review protocol was registered or published. The completed PRISMA‐ScR checklist is provided in Supplementary S1.

### 2.2 Eligibility Criteria

Eligibility criteria were developed using the Population–Concept–Context framework, as recommended for scoping reviews (Peters et al. 2020; Peters et al. 2022). The population comprised service providers working in paediatric disability‐related services. The concept comprised identifiable and structured frameworks, models, programmes, approaches or tools designed to translate family‐centred principles into practice, together with service providers' perspectives and experiences of their application or implementation. The context included paediatric rehabilitation, early intervention, children's disability and other relevant health, education or community services for children and young people aged 0–18 years and their families.

For eligibility assessment, an approach was considered an FCP framework when it: (1) was presented in the study as family‐centred or as supporting the application of family‐centred principles; (2) included at least one identifiable element that structured practice, such as stated principles, defined intervention components, a sequence of activities, decision‐making steps, or a tool or protocol guiding assessment, goal setting, intervention planning or service delivery; and (3) involved service providers in its application, delivery or implementation. Studies were also required to report empirical data concerning service providers' views, perceptions, experiences, understanding or reflections arising from the use or implementation of the approach. Studies discussing FCP only as a general philosophy, without examining an identifiable approach and its application in practice, were excluded. The full eligibility criteria are presented in Table 1.

**TABLE 1:** Eligibility criteria according to the PCC framework.

PCC component/criterion	Inclusion criteria	Exclusion criteria
Population	Service providers working in children's disability, paediatric rehabilitation, early intervention or related paediatric disability services. Service providers included allied health professionals, therapists, physicians, nurses, psychologists, social workers, early intervention practitioners, service coordinators and other relevant professionals.	Studies reporting only children's or family members' perspectives without service provider data; studies focused exclusively on adult disability services.
Concept	Identifiable and structured family‐centred frameworks, models, programmes, approaches or tools containing principles, components, procedures or processes that guided service planning, delivery or implementation and that had been applied or implemented by service providers in practice. Studies were required to report service providers' perspectives or experiences of applying or implementing the approach.	Studies discussing family‐centred practice only as a general philosophy or concept; studies examining an approach without identifiable principles, components, procedures or processes guiding practice; studies in which the approach had not been applied or implemented by service providers; and studies that did not report service providers' perspectives or experiences of its application or implementation.
Context	Paediatric disability‐related services for children and young people aged 0–18 years and their families, including paediatric rehabilitation, early intervention, hospital, primary‐care, community‐based, home‐based, education‐related and teleintervention services.	Studies conducted outside paediatric disability‐related service contexts or studies in which findings relating to paediatric disability services could not be separately identified.
Publication type	Full‐text empirical journal articles reporting qualitative, quantitative or mixed‐methods research.	Grey literature, theses, dissertations, reports, conference abstracts, protocols, reviews, editorials, commentaries and opinion papers.
Language	English.	Studies published in languages other than English.
Study design	Qualitative, quantitative or mixed‐methods empirical studies.	Non‐empirical papers or studies without relevant empirical data.

### 2.3 Information Sources and Search Strategy

A structured search strategy was developed from the review question and PCC framework and refined iteratively to improve relevance and conceptual clarity (Levac et al. 2010; Peters et al. 2020). The final strategy combined five concept groups relating to service providers; FCP, children and young people; disability‐related conditions; and perspectives. Synonyms within each concept group were combined using OR, and the five groups were combined using AND.

Searches were conducted in CINAHL Complete, APA PsycINFO, SocINDEX with Full Text, Academic Search Complete, Scopus and MEDLINE via PubMed. The final searches were conducted on 29 January 2026, and no date limits were applied. Reference lists of included studies were also screened to identify additional relevant articles. Full search strategies and database‐specific adaptations are provided in Supplementary S2.

### 2.4 Study Selection

All records identified through database searching were exported to EndNote 20 (Clarivate 2020), where duplicate records were removed. The deduplicated records were then imported into Rayyan for screening (Ouzzani et al. 2016). Titles and abstracts were screened against the predefined eligibility criteria, and full texts were retrieved for records that appeared potentially relevant. Full‐text articles were assessed for eligibility, and reasons for exclusion were recorded at this stage, consistent with PRISMA‐ScR reporting guidance (Tricco et al. 2018).

During full‐text screening, approaches identified through the broader FCP search terms were assessed against the operational definition. Studies were eligible when the approach was identifiable and structured, had been applied by service providers and generated empirical evidence of providers' perspectives or experiences. Screening was conducted by one reviewer, with repeated checking against these criteria.

### 2.5 Data Charting

Data were charted using a structured data‐charting form developed for this review. Extracted data included study characteristics, service setting, participant characteristics, professional background, child population, the name and type of FCP framework, its principal components or processes, how it was applied or implemented by service providers and findings concerning service providers' perspectives and experiences. These findings included perceived usefulness, benefits, limitations, barriers, facilitators, training and support needs and resource requirements. Data charting was conducted by one reviewer. The data‐charting form was refined during the review process, consistent with guidance that data charting in scoping reviews may be iterative (Peters et al. 2022; Peters et al. 2021).

### 2.6 Data Analysis and Synthesis

Data were synthesised descriptively and narratively, in line with the mapping purpose of scoping reviews (Arksey and O'Malley 2005; Levac et al. 2010; Peters et al. 2022). Descriptive summaries mapped publication year, country, study design, service setting, professional groups, child populations and the FCP frameworks identified. The frameworks were grouped according to their principal characteristics and functions in practice. Findings relating to service providers' perspectives and experiences were grouped thematically according to similarities and differences across studies, focusing on framework application, perceived usefulness, professional role changes, support needs, resource implications, barriers, facilitators and contextual influences.

### 2.7 Quality Appraisal

Formal methodological quality appraisal was not undertaken because the aim of this scoping review was to map the extent, range and nature of available evidence rather than to assess intervention effectiveness or exclude studies on the basis of methodological quality (Peters et al. 2022; Tricco et al. 2018). Consequently, the findings describe the range of reported perspectives and experiences but should not be interpreted as establishing the effectiveness of particular FCP frameworks or the strength of evidence supporting them.

## 3 Results

### 3.1 Study Selection

A total of 3140 records were identified through database searching. After the removal of 1105 duplicates, 2035 records were screened by title and abstract, and 1984 records were excluded. Fifty‐one reports were sought for retrieval, of which two could not be retrieved. Forty‐nine full‐text articles were assessed for eligibility, and 28 were excluded because they did not focus on FCP frameworks (*n* = 15), focused on service quality or service comparison without examining a framework (*n* = 7), were not conducted in paediatric disability‐related settings (*n* = 3) or did not clearly report service providers' perspectives (*n* = 3). In total, 21 studies were included in the review. The study identification, screening and inclusion process is presented in Figure 1.

**FIGURE 1:**
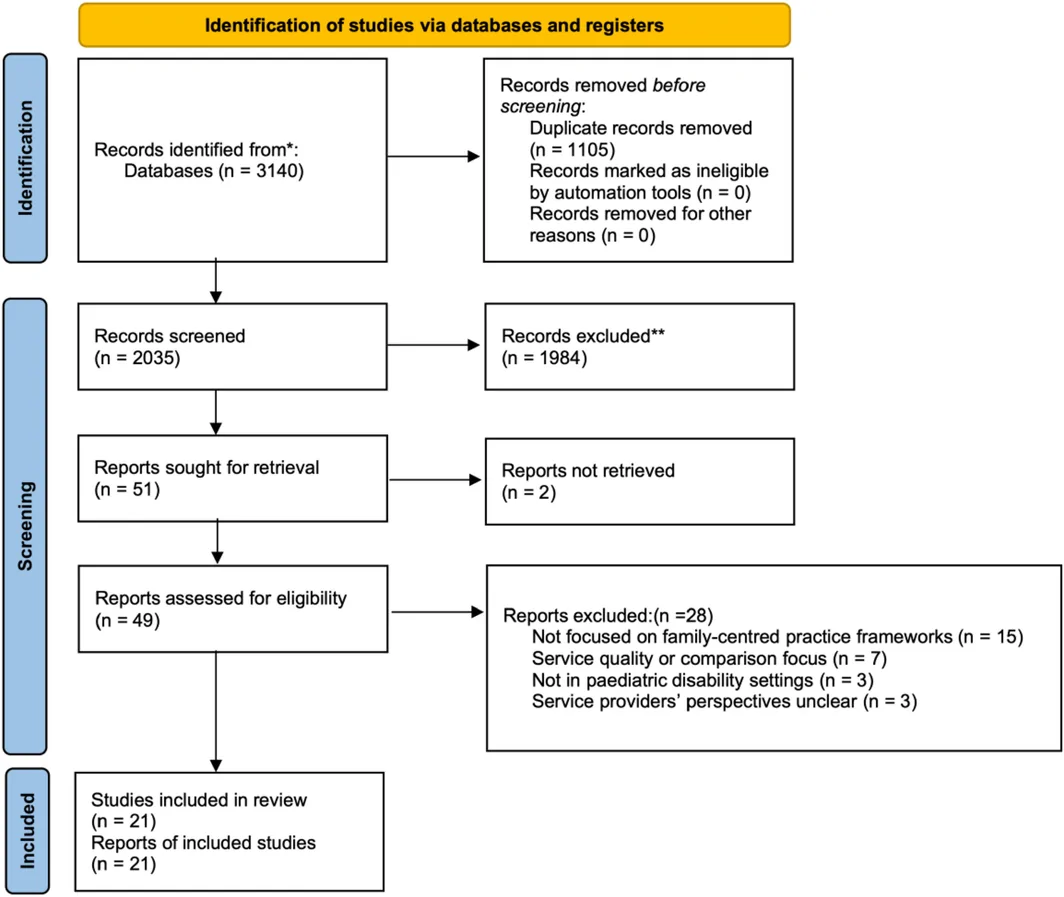
PRISMA flow diagram.

### 3.2 Characteristics of Included Studies

The 21 included studies were published between 2005 and 2025 and were conducted across nine countries or regions. Studies were conducted in the United States (*n* = 7), Australia (*n* = 4), Canada (*n* = 3), the United Kingdom (*n* = 2) and Ireland, the Netherlands, the United Arab Emirates, China (Taiwan) and New Zealand (*n* = 1 each). Thirteen studies used qualitative designs, five used mixed methods and three used quantitative designs. Study characteristics are presented in Table 2.

**TABLE 2:** Summary of characteristics of included studies.

Study	Country	Setting	Participants	Child population	Framework	Design
MacKean et al. (2005)	Canada	Developmental services at a children's hospital in Alberta, Canada.	16 frontline healthcare providers 4 speech‐language pathologists, 4 developmental paediatricians and 8 from other disciplines including nursing, social work, occupational therapy, physical therapy and psychology	Children with developmental problems (autism, down syndrome); age not specified but part of a preschool treatment program (approx. 0–5 years)	Family‐Centred Care (FCC) conceptual framework.	Qualitative (grounded theory study using focus groups).
Pennington and Thomson (2007)	United Kingdom	Various National Health Service (NHS) Trusts in the United Kingdom (primarily Northern England) serving rural, semirural and urban populations.	16 speech and language therapists (SLTs)	Young children with motor disorders (e.g., cerebral palsy); preschool age (under 5)	It Takes Two to Talk – The Hanen Program	Qualitative (focus groups)
Carbone et al. (2010)	United States	Primary care paediatrician offices in a large metropolitan area in Salt Lake City, Utah, USA.	9 Primary care paediatricians.	Children with autism spectrum disorders (asds); Sample ages were 4, 5, 6, 7, 11 and 14 years old	Medical Home Model.	Qualitative (focus groups).
Rodger et al. (2012)	Australia	A community‐based Family and Early Childhood Service (FECS) provided by Disability Services, Department of Communities in Queensland, Australia.	10 team members (a multidisciplinary team including 2 occupational therapists, 2 speech and language pathologists, 1 physiotherapist, 1 psychologist, 2 social workers, and 2 programme officers).	Children aged up to 6 years with global developmental delay or cognitive impairment, up to 6 years old	Family Goal Setting Tool (FGST)	Qualitative (focus groups and individual interviews).
Khetani et al. (2015)	United States	A community‐based service agency funded to promote full community inclusion	7 community‐based agency staff Supervisors (*n* = 1), coordinators (*n* = 3) and class leaders (*n* = 3)	Children with disabilities ages 5–17.	Decisional Support Algorithm (used with the Participation and Environment Measure for Children and Youth, PEM‐CY)	Mixed methods (explanatory sequential)
Carroll and Sixsmith (2016)	Ireland	One specific Early Intervention (EI) team in Ireland that was part of a non‐governmental organisation. The study involved teams in both urban and rural regions.	17 professionals: A multidisciplinary team consisting of: 4 nurses, 3 speech and language therapists (SLTs), 3 physiotherapists, 2 occupational therapists (OTs), 1 social worker, 1 care assistant, 1 family support worker, 1 dietician, 1 psychologist and 1 team leader	Children (birth to 5) with developmental/intellectual disabilities (Down syndrome, etc.). Birth to 5 years (child participants were aged 2 to 5 years)	Trajectory of Relationship Development	Qualitative case study (grounded theory).
Lavigne et al. (2017)	Canada	A paediatric rehabilitation centre (Centre de réadaptation Marie Enfant) in Montreal, Canada, specifically within the Cerebral Motor Deficits (CMD) program.	10 members of a cerebral motor deficits (CMD) team (2 occupational therapists, 2 physical therapists, 1 speech therapist, 1 paediatrician, 1 social worker, 1 specialised educator, 1 psychologist and 1 clinical coordinator).	Children aged 2 years and younger with cerebral motor deficits (associated with a GMFCS score of 4 or 5)	Préparation au plan d'intervention (PAPI), version 2.1	Qualitative (focus group).
Graham et al. (2018)	New Zealand	Child development and rehabilitation services in New Zealand. Services were a mix of publicly funded community services, charity‐funded centre‐based services, and special schools.	16 therapists (12 occupational therapists and 4 physiotherapists)	Children with single or multiple impairments (e.g., autism, visual impairment, neurodevelopmental disorders), aged 3–15 years.	Occupational Performance Coaching (OPC).	Qualitative (thematic analysis)
van Maren‐Suir et al. (2018)	Netherlands	A rehabilitation centre in the Netherlands, specifically implementing consultations within the family home.	7 service providers (one from each discipline: paediatric physiatrists, physician assistants, speech therapists, occupational therapists, physical therapists, social workers, and paediatric psychologists)	Children aged 0–5 years with Mixed diagnoses including cerebral palsy, developmental delay, or syndromes.	@home system	Qualitative (iterative implementation process).
Jones et al. (2019)	Australia	An autism‐specific Early Intervention (EI) service (Autism Queensland) in Australia, covering both metropolitan and regional centres.	11 practitioners (a multidisciplinary group including 2 occupational therapists (OT), 5 teachers (T), 1 family support worker (FSW), and 3 speech‐language pathologists (SLP)).	Children with autism spectrum disorder (ASD); Preschool age (child participants were aged 3–6 years)	Family Goal Setting Tool: Autism Spectrum Disorder Version (FGST: ASD Version)	Qualitative (inductive content analysis)
Schwellnus et al. (2020)	Canada	A children's treatment centre in Ontario, Canada, where services were provided in outpatient clinics and in community settings such as homes, daycares and schools.	6 Experienced therapists (4 occupational therapists and 2 physical therapists)	Children and youth with chronic health conditions and various neurodevelopmental disabilities; children and youth (typically birth through transition to adulthood).	Solution‐Focused Coaching in paediatric rehabilitation (SFC‐peds)	Qualitative (In‐depth semistructured interviews).
Popova et al. (2022)	United States	An Early Intervention (EI) program in Illinois, USA.	101 therapists (developmental, occupational, physical and speech therapists)	Mixed developmental delays/disabilities Infants and toddlers younger than 36 months receiving EI services	Intentional Relationship Model (IRM).	Quantitative: Prospective descriptive study (cross‐sectional)
Callanan et al. (2023)	Australia	An Early Childhood Development Service (Carpentaria Kids) in the Northern Territory, Australia	14 allied health professionals (4 occupational therapists, 2 physiotherapists, 3 psychologists, and 5 speech pathologists).	Children with developmental delays; context is early childhood development (approx. birth to age 5)	Parent Child Relationally Informed – Early Intervention (PCRI‐EI)	Mixed methods (survey with Likert and open‐ended items).
Opoku et al. (2024)	United Arab Emirates	Various health facilities, rehabilitation centres, and schools in the Emirate of Abu Dhabi, United Arab Emirates. The study included participants from different regions including Abu Dhabi city and Al Ain.	150 allied health professionals Allied health professionals, including physiotherapists, speech and language pathologists (SLPs), psychologists, psychiatrists, and occupational therapists (OTs)	Children receiving disability diagnosis and ongoing support; ages across 0–12 months, 12–36 months, 4–10 years, and 11–18 years	Family Centered Care (FCC) Model.	Quantitative (confirmatory factor analysis and MANOVA).
Fabrizi et al. (2024)	United States	Parks and community centres in a two‐county region in the Southeastern United States.	19 EI facilitators (multidisciplinary facilitators including 2 occupational therapists, 2 physical therapists, 2 speech‐language pathologists, 1 mental health counsellor, and 12 infant‐toddler developmental specialists).	Children at‐risk or with developmental delay; Sample ages 12 to 60 months (M = 35 months)	SEE Me (Social Emotional Education) Therapeutic Playgroup (TPG)	Mixed methods (single group pre/post).
Shie et al. (2025)	China (Taiwan)	Two paediatric occupational therapy internship programs located in outpatient medical hospitals in Taiwan	49 undergraduate occupational therapy interns	Children under 6 years old diagnosed with developmental delays or disabilities.	Family‐Centered Teaching (FACT) Model	Quantitative: Two‐group quasi‐experimental design.
Kuhn and Higgins (2025b)	United States	Early Intervention (EI) programs across a Midwestern U.S. state.	12 EI service providers early childhood special educators (ECSE), speech‐language pathologists (SLP), occupational therapists (OT), physical therapists (PT) and 7 independent, dedicated service coordinators (SCs).	20 children (mean age = 33.55 months) with developmental delays or disabilities.	Getting Ready Approach	Interpretive qualitative study (interviews and document reviews of action plans)
Kuhn and Higgins (2025a)	United States	Early Intervention (EI) programs across a Midwestern U.S. state. The participants were recruited from urban, suburban, and rural settings.	Survey: 50 trained SCs, 15 supervisors, and 81 EI providers Focus groups: 41 participants (21 SCs, 11 supervisors, 9 EI providers) Dedicated early intervention service coordinators (SCs), SC supervisors, and EI providers (educators, PTs, OTs, SLPs)	Infants and toddlers with verified delays or disabilities (birth to age 3)	Getting Ready Approach (adapted for SCs)	Mixed‐method sequential explanatory design (surveys followed by focus group interviews)
Wilson et al. (2025)	Australia	Paediatric rehabilitation settings in Australia, primarily a tertiary children's hospital and private clinics in Melbourne.	9 Paediatric rehabilitation specialists (occupational therapists, physical therapists, speech pathologists, paediatric medical specialists, social workers, and data analysts).	Children with disabilities (cerebral palsy, brain injury); sample includes parents of children aged 6, 17 and 18 years old	Goal sharing digital platform (innovation‐based digital health product)	Qualitative (interviews and workshops)
Massey et al. (2025)	United Kingdom	A National Health Service (NHS) outpatient rehabilitation service in a tertiary children's hospital in London, UK and community settings.	8 Occupational therapists (3 hospital‐based and 5 community‐based).	Children with Hemiplegia (Unilateral CP) aged 16 months to 4 years	Codelivering Intensive Upper Limb Intervention	Qualitative (thematic analysis using theoretical domains framework).
Harbin et al. (2025)	United States	Teleintervention sessions (virtual visits) conducted in the child's natural home environment	4 early intervention practitioners (2 special educators, 1 physical therapist and 1 speech‐language pathologist).	Children identified as at‐risk or diagnosed with developmental delays (e.g., Global Developmental Delay) (age group 12 months to 2 years old)	PIWI (Parents Interacting with Infants) Triadic Strategies	Mixed methods (single‐case research design + qualitative interviews).

Included studies spanned early intervention, paediatric rehabilitation, hospital, community, primary‐care, home‐based and teleintervention services. Participants included allied health professionals, physicians, nurses, psychologists, social workers, educators, service coordinators and multidisciplinary teams. Child populations included cerebral palsy, autism, developmental delay, intellectual disability, neurodevelopmental disabilities, hemiplegia and mixed or complex profiles. Most focused on early childhood or preschool ages; some included older children or adolescents (Table 2).

### 3.3 FCP Frameworks Identified

The included approaches were grouped by primary function into five categories: conceptual and guiding frameworks, service delivery models, coaching and relational approaches, intervention and training models and assessment and collaborative planning tools. The categories were used for descriptive mapping and were not mutually exclusive. Approaches serving more than one function were classified according to their main use in the included study.

Conceptual and guiding frameworks described the principles, relationships or service characteristics underpinning FCP, including family centrality, partnership, respectful treatment and information provision (MacKean et al. 2005; Opoku et al. 2024). Service delivery models structured how or where services were provided, ranging from home consultations and codelivered interventions to the organisation of coordinated primary care (Carbone et al. 2010; Massey et al. 2025; van Maren‐Suir et al. 2018).

Coaching and relational approaches guided interactions between providers and families through reflective conversations, responsiveness, collaborative problem‐solving and caregiver coaching (Callanan et al. 2023; Carroll and Sixsmith 2016; Graham et al. 2018; Harbin et al. 2025; Popova et al. 2022; Schwellnus et al. 2020). Intervention and training models used structured teaching, facilitated activities or supervised practice to develop caregiver or provider competencies (Fabrizi et al. 2024; Kuhn and Higgins 2025a, 2025b; Pennington and Thomson 2007; Shie et al. 2025).

Assessment and collaborative planning tools provided defined processes for identifying family priorities and translating them into goals, decisions or service plans (Jones et al. 2019; Khetani et al. 2015; Lavigne et al. 2017; Rodger et al. 2012; Wilson et al. 2025). Table 3 presents each approach, its defining components and its application by service providers.

**TABLE 3:** Overview of family‐centred practice frameworks identified in the included studies.

Framework	Description used in the included study	Primary function in this review	Defining components or processes	Application by service providers
@home system (van Maren‐Suir et al. 2018)	Home consultation service model	Service delivery model	Three forms of home consultation: observation, advice and therapy	Rehabilitation professionals used home visits to assess children in everyday settings, respond to family questions and provide intervention in the home
Codelivered intensive intervention (Massey et al. 2025)	Distributed parent–therapist intervention model	Service delivery model	Parent‐led home sessions combined with hospital‐ and community‐therapist sessions	Therapists trained and supported parents to deliver intensive upper‐limb intervention across home and clinical settings
Decisional Support Algorithm (Khetani et al. 2015)	Four‐step decision‐support algorithm used with the PEM‐CY	Assessment and collaborative planning tools	Parent ranking of activities, consideration of child preferences, goal clarification and identification of environmental supports and barriers	Providers used the structured process with families to interpret assessment findings, prioritise participation goals and develop intervention strategies
Family Goal Setting Tool (FGST) (Rodger et al. 2012)	Visual card‐sorting goal‐setting tool	Assessment and collaborative planning tools	Seventy‐eight cards covering child and family priorities, sorted into “yes”, “no” and “maybe” categories	Team members used the cards with families to identify, discuss and prioritise child‐ and family‐level goals
Family Goal Setting Tool (FGST‐ASD) (Jones et al. 2019)	Autism‐specific visual card‐sorting tool	Assessment and collaborative planning tools	Goal cards organised across eight domains and sorted to identify immediate and future priorities	Practitioners supported families to identify and prioritise three to six goals relevant to the child and family
Family‐Centered Teaching (FACT) Model (Shie et al. 2025)	Structured teaching and supervised practice model	Intervention and training model	Didactic teaching in empathy, communication, assessment and goal setting, followed by supervised practice with families	Occupational therapy interns gathered family information, identified parental priorities, developed goals and implemented intervention collaboratively under supervision
Family‐Centred Care (FCC) Model (Opoku et al. 2024)	FCC model operationalised through the MPOC‐SP	Conceptual and guiding framework	Respectful treatment, interpersonal sensitivity, provision of child‐specific information and provision of general information	Professionals applied these domains in service delivery, and their reported use of family‐centred practices was assessed using the MPOC‐SP
FCC conceptual framework (MacKean et al. 2005)	Conceptual framework developed from family and provider experiences	Conceptual and guiding framework	Family centrality, recognition of family uniqueness and parent expertise, competency enhancement, collaboration and family networks	Providers used the principles to understand family–professional relationships and examine differences between FCC policy and practice
Getting Ready Approach (adapted) (Kuhn and Higgins 2025a)	Professional‐development framework	Intervention and training model	Eight interaction strategies addressing relationships, joint decision‐making, family competence, information sharing, observation and guided practice	Service coordinators and early‐intervention providers used the strategies during home visits, supported by training, coaching and fidelity feedback
Getting Ready Approach (Kuhn and Higgins 2025b)	Adapted evidence‐based professional‐development approach	Intervention and training model	Seven interaction strategies and a structured home visit guide covering opening, main agenda and closing/reflection	Service coordinators used the guide to structure home visits, strengthen partnerships, share resources and document agreed actions
Goal‐sharing digital platform (Wilson et al. 2025)	Purpose‐built family‐centred digital health platform	Assessment and collaborative planning tools	Shared goal records, asynchronous communication, progress monitoring, video and resource sharing, and printable documentation	Providers used the platform with families to establish, share and monitor goals across services and settings
Hanen Programme (Pennington and Thomson 2007)	Structured parent‐training programme	Intervention and training model	Eight group sessions and three individual video‐based home‐coaching visits	Speech and language therapists trained and coached parents to recognise communication signals and use responsive interaction strategies
Intentional Relationship Model (IRM) (Popova et al. 2022)	Interpersonal model for the therapeutic use of self	Coaching and relational approach	Six therapeutic modes: advocating, collaborating, encouraging, empathising, instructing and problem‐solving	Therapists selected and adapted interpersonal modes when communicating with families and responding to their needs
Medical Home Model (Carbone et al. 2010)	Family‐centred model of primary‐care delivery	Service delivery model	Accessible, continuous, comprehensive, coordinated, compassionate, family‐centred and culturally effective care	Paediatricians used the model to guide care coordination, communication, shared decision‐making and links with other services
Occupational Performance Coaching (OPC) (Graham et al. 2018)	Strengths‐based coaching intervention	Coaching and relational approach	Reflective questioning, collaborative goal setting, problem‐solving and action–reflection processes	Therapists used coaching conversations to help caregivers identify goals, develop strategies and apply them within everyday routines
PAPI (Lavigne et al. 2017)	Structured family‐needs assessment questionnaire	Assessment and collaborative planning tools	Interview‐based assessment covering health, autonomy, communication, relationships, leisure, participation and education	Professionals administered the questionnaire with families to identify priorities, prepare intervention plans and coordinate multidisciplinary input
PCRI‐EI (Callanan et al. 2023)	Manualised relational model of practice	Coaching and relational approach	Strategies for engagement, responsiveness, family empowerment, parent well‐being and reflective practice	Therapists used the manual, case examples and reflective supervision to strengthen responsiveness and therapeutic relationships with parents and children
PIWI Triadic Strategies (Harbin et al. 2025)	Six practitioner strategies for caregiver coaching	Coaching and relational approach	Establishing the dyadic context, affirming parent competence, focusing attention, providing developmental information, modelling and suggesting	Early‐intervention practitioners used the six strategies during teleintervention to coach caregivers within caregiver–child interactions
SEE Me Therapeutic Playgroup (Fabrizi et al. 2024)	Structured therapeutic playgroup programme based on PIWI	Intervention and training model	Eight semistructured group sessions using play activities, facilitator guides, parent resources and triadic strategies	Multidisciplinary facilitators coached parents during play activities and supported parent–child interaction and child participation
Solution‐Focused Coaching (SFC‐peds) (Schwellnus et al. 2020)	Strengths‐based coaching model	Coaching and relational approach	Solution‐building questions, positive feedback, goal setting and action–reflection cycles	Therapists used coaching conversations to support family‐defined goals, caregiver problem‐solving and application of strategies in everyday environments
Trajectory of Relationship Development (Carroll and Sixsmith 2016)	Five‐stage relationship development model	Coaching and relational approach	Initiating, experimenting, intensifying, integrating and transitioning stages	Early‐intervention professionals used the stages to understand and guide the development, maintenance and transition of relationships with families

### 3.4 Service Providers' Perspectives on FCP Frameworks

Six themes were identified from findings concerning service providers' perspectives and experiences of applying or implementing FCP frameworks: professional role transformation, relational and communication competencies, collaborative goal setting in practice, barriers to implementation, training and professional support needs and the influence of context and service delivery. The synthesis included provider quotations, provider‐derived findings, measured provider findings and joint provider–family findings where providers were explicitly included; evidence type is identified in Table 4.

**TABLE 4:** Thematic synthesis of service providers' perspectives with illustrative evidence.

Theme	Subtheme	Illustrative evidence	Evidence type	Supporting studies
1. Professional role transformation	From expert to facilitator	“I love that solution focused coaching … that I do not necessarily need to be the expert, I can bring some knowledge, but … but I'm not the expert. You know, be the client, that's the expert.”	Provider quotation	Schwellnus et al. (2020)
		“The therapist is no longer the expert in the room (so what are parents paying for?)”	Provider quotation	Callanan et al. (2023)
		Interns demonstrated a significant increase in “treating caregivers as the experts to discuss their opinions.”	Measured provider finding	Shie et al. (2025)
		“The role of the clinician was re‐conceptualised as a collaborative facilitator.”	Provider‐derived finding	Graham et al. (2018)
		“Adopting a totally different approach … giving themselves space to actually watch and see what the children are doing.”	Provider‐derived finding	Pennington and Thomson (2007)
		“We want to provide enough information for parents so that they feel empowered around making decisions for care for their child.”	Provider quotation	MacKean et al. (2005)
		Practitioners described moving away from a “problem‐oriented, therapist‐directed approach.”	Provider‐derived finding	Graham et al. (2018)
	Biting one's tongue	“Being a careful … listener demands a considerable amount of self‐regulation to ‘bite my tongue’.”	Provider‐derived finding	Schwellnus et al. (2020)
		Practitioners report it is “hard to sit on my hands” and resist the habit of providing immediate clinical advice.	Provider‐derived finding	Graham et al. (2018)
		Results showed a “marked decrease in directive strategy use” such as unsolicited suggestions.	Measured provider finding	Harbin et al. (2025)
		“I have to be quiet and I have to bite my tongue … being present and waiting and being patient.”	Provider quotation	Schwellnus et al. (2020)
	Emotional responses to change	The shift to a non‐expert role was conceived as “liberating” and “freeing” as it reduced the pressure to have all the answers.	Provider‐derived finding	Schwellnus et al. (2020)
		“Initially it felt like I wasn't doing anything … supervisors … would wonder what the heck I was doing.”	Provider quotation	Schwellnus et al. (2020)
		Transitioning to coaching via teleintervention was described as “nerve‐wracking” due to the perceived lack of physical control.	Provider‐derived finding	Harbin et al. (2025)
		“Initial trust in the process … seemed ‘airy fairy’ at first.”	Provider‐derived finding	Callanan et al. (2023)
2. Relational and communication competencies	Prioritising relationship over information	“Therapists used family‐centred practices specific to relationship building more frequently than information sharing.”	Measured provider finding	Popova et al. (2022)
		“Now I can sit with the emotions [of parents] a lot better than I have done in the past.”	Provider quotation	Callanan et al. (2023)
		Relationship development is described as an “overlapping five‐stage developmental trajectory.”	Provider‐derived finding	Carroll and Sixsmith (2016)
		Understanding child's experience helps the therapist formulate a better “uniqueness of the child rather than just focusing on diagnostic info.”	Provider‐derived finding	Callanan et al. (2023)
	Active listening and empathy	The overarching theme of “Listening better” was found to be the foundation for successful coaching.	Provider‐derived finding	Graham et al. (2018)
		“Therapists reported that they most frequently used the empathising and encouraging modes.”	Measured provider finding	Popova et al. (2022)
		Essential to have “conversations and listening to families' needs” to identify meaningful goals.	Provider‐derived finding	Wilson et al. (2025)
		“OPC is more around ‘okay, I'm going to come and sit beside you and then come look and see what the world looks like from your direction.’”	Provider quotation	Graham et al. (2018)
	Building equal partnerships	“Parents and service professionals … perceived a more equal partnership” during home‐based consultations.	Mixed provider and family finding	van Maren‐Suir et al. (2018)
		Teams used PAPI to promote “shared decision making between the families and the team.”	Provider‐derived finding	Lavigne et al. (2017)
		Shared communication about a client's goals was identified as the “glue” that kept professionals together.	Provider‐derived finding	Carroll and Sixsmith (2016)
3. Collaborative goal setting in practice	Using practical tools to support collaboration	“Families gained a lot of power in the meeting, rather than coming to us and saying, ‘whatever you think will be fine’.”	Provider quotation	Jones et al. (2019)
		Visual card‐sort tools “demonstrated the potential to overcome barriers related to communication and literacy levels.”	Provider‐derived finding	Rodger et al. (2012)
		“Rank ordering is something that you could see would be feasible to help a family figure out where to start.”	Provider quotation	Khetani et al. (2015)
		The PAPI tool allowed the multidisciplinary team to “refocus on parents' priorities” when medical needs became overwhelming.	Provider‐derived finding	Lavigne et al. (2017)
	Defining meaningful, real‐life goals	“They need to be meaningful and achievable for that young person, even though there may be quite a lot of steps to actually getting to that point.”	Provider quotation	Wilson et al. (2025)
		Goal setting became more “tangible and concrete” by putting “who does what” down on paper.	Provider‐derived finding	Kuhn and Higgins (2025b)
		“We set these goals, you know, they are not just dead goals, they are live ones, how are we going with them?”	Provider quotation	Graham et al. (2018)
	Digital support for collaborative goal setting	Digital platforms facilitate “timely, asynchronous communication” which fits better into busy family lives.	Provider‐derived finding	Wilson et al. (2025)
		Mobile technology mitigates provider concerns about the “feasibility of consistent evaluation in busy practices.”	Provider‐derived finding	Khetani et al. (2015)
4. Barriers to implementation in practice	Limited time and high workload	“Lack of time” was identified as the primary barrier to coordinating care and providing a medical home.	Provider‐derived finding	(Carbone et al. (2010)
		“Professionals with increasing caseloads and administration duties may lack the time necessary to build relationships.”	Provider‐derived finding	Carroll and Sixsmith (2016)
		“Traffic congestions meant that service providers had insufficient time left for the actual home consultations.”	Provider‐derived finding	van Maren‐Suir et al. (2018)
		“Therapists … are already under the pump … [they] might find [digital tools] problematic.”	Provider quotation	Wilson et al. (2025)
	Tension with the medical model	“Some people feel quite medical model … and just want to cut to the chase and get it done.”	Provider quotation	Schwellnus et al. (2020)
		One senior educator felt that holistic family goals were “outside the scope of her educational role.”	Provider‐derived finding	Jones et al. (2019)
		“It was hard to untrain the habits learned and polished over the years doing therapy TO children.”	Provider quotation	Callanan et al. (2023)
		Some therapists felt “undermined” by colleagues who were still “prepared to give the answers.”	Provider‐derived finding	Graham et al. (2018)
	Challenges in sharing information and resources	“Providing general information [community resources] was rated the lowest” by therapists in multiple studies.	Measured provider finding	Popova et al. (2022)
		Health professionals “struggle to engage with the wider issues beyond medical treatment.”	Provider‐derived finding	Opoku et al. (2024)
		“Paediatricians acknowledged their lack of awareness of community resources … [families] know more than I know.”	Provider quotation	Carbone et al. (2010)
5. Training and professional support needs	Gaps in professional training	Relational skills needed for FCP “are not included in their undergraduate education.”	Provider‐derived finding	Callanan et al. (2023)
		“My [graduate] program did not really focus on … coaching … we model for the child and make suggestions.”	Provider quotation	Harbin et al. (2025)
		“Broad aspects of family‐centred care do not commonly form part of professional training programmes.”	Provider‐derived finding	Opoku et al. (2024)
	Value of mentoring and supervision	“Reflective supervision” was rated as the most valued support (4.79/5) for clinicians transitioning to FCP.	Measured provider finding	Callanan et al. (2023)
		Training was most effective when it included “individual virtual meetings with a state‐approved coach” and video feedback.	Provider‐derived finding	Kuhn and Higgins (2025a)
		“Therapists identified various education and training needs to improve their capabilities to coach parents.”	Provider‐derived finding	Massey et al. (2025)
	Developing coaching skills	“Giving the providers that opportunity to learn how to facilitate … will help them be better coaches.”	Provider quotation	Fabrizi et al. (2024)
		Coaching training leads to a “parental shift of narrative from bad to struggling child.”	Provider‐derived finding	Callanan et al. (2023)
		“Therapists should be skilled in education and coaching when supporting parents.”	Provider‐derived finding	Massey et al. (2025)
6. Influence of context and service delivery	Power balancing in home contexts	“Home consultations provide a good natural therapeutic environment where a child can be itself.”	Mixed provider and family finding	van Maren‐Suir et al. (2018)
		“I think parents feel more at ease to speak about their daily routine when they are at home than when they are outside and I think that it's a trust relationship that begins at this moment.”	Provider quotation	Lavigne et al. (2017)
		“In home consultations, the therapists are visitors, instead of the parents being visitors at the rehabilitation centre.”	Provider‐derived finding	van Maren‐Suir et al. (2018)
		“The clinic environment did not always lend itself to enabling parents to lead or codeliver.”	Provider‐derived finding	Massey et al. (2025)
	Complexities of remote delivery	The “benefit” of teleintervention is that caregivers “have to be the ones doing it” because the therapist cannot reach through the screen.	Provider‐derived finding	Harbin et al. (2025)
		“Virtual sessions shifted the dynamic and made it easier for the parent to lead the session.”	Provider‐derived finding	Massey et al. (2025)
		Modelling strategies through a screen was described as “much more challenging” than in‐person.	Provider‐derived finding	Harbin et al. (2025)
	Interprofessional team collaboration	“Shared training… facilitated professional collaboration to cover dual aims of the home visit.”	Provider‐derived finding	Kuhn and Higgins (2025b)
		“It was difficult to keep activities going while coaching parents and completing paperwork in the 1 h timeframe” of a playgroup.	Provider quotation	Fabrizi et al. (2024)
		“Teams found that shared communication about a client's goals was the ‘glue’ that kept them together.”	Provider‐derived finding	Carroll and Sixsmith (2016)

#### 3.4.1 Professional Role Transformation

Service providers frequently described FCP as requiring a shift from being the primary expert or decision‐maker towards becoming a facilitator, coach or collaborative partner (Callanan et al. 2023; Graham et al. 2018; Schwellnus et al. 2020). This involved recognising parents and caregivers as experts in their child's everyday life, providing information that supported informed family decision‐making and maintaining a professional role in guidance and shared planning (MacKean et al. 2005; Schwellnus et al. 2020; Shie et al. 2025).

Several studies showed that this role shift required professionals to reduce directive behaviours and create space for family‐led problem‐solving (Graham et al. 2018; Harbin et al. 2025; Schwellnus et al. 2020). Service providers described this as requiring conscious self‐regulation, particularly when they felt an impulse to provide immediate advice or take control of the intervention process (Graham et al. 2018; Schwellnus et al. 2020). Although some professionals experienced this shift as freeing because it reduced the pressure to provide all solutions, others described uncertainty, discomfort or anxiety when moving away from familiar therapist‐led roles (Callanan et al. 2023; Harbin et al. 2025; Schwellnus et al. 2020).

#### 3.4.2 Relational and Communication Competencies

Relational and communication competencies were central to how service providers enacted FCP across studies (Callanan et al. 2023; Carroll and Sixsmith 2016; Graham et al. 2018; Popova et al. 2022). Service providers emphasised listening, empathy, emotional responsiveness and trust‐building as mechanisms for understanding family priorities and developing collaborative relationships (Callanan et al. 2023; Graham et al. 2018; Popova et al. 2022).

Relationship‐building shaped whether families could express concerns, identify priorities and participate in planning (Carroll and Sixsmith 2016; Graham et al. 2018; Wilson et al. 2025). Providers reported that home‐based consultations and structured assessment processes could support communication, trust and more equal partnerships by allowing professionals to understand family routines and priorities within everyday contexts (Lavigne et al. 2017; MacKean et al. 2005; van Maren‐Suir et al. 2018).

#### 3.4.3 Collaborative Goal Setting in Practice

Collaborative goal setting was a key way in which FCP was operationalised in practice (Jones et al. 2019; Khetani et al. 2015; Rodger et al. 2012; Wilson et al. 2025). Structured tools, including card‐sorting approaches, needs assessment tools and participation‐based decision‐support processes, helped families identify priorities and supported professionals to translate family concerns into concrete intervention goals (Jones et al. 2019; Khetani et al. 2015; Lavigne et al. 2017; Rodger et al. 2012).

Service providers valued goals that were meaningful in everyday life rather than limited to impairment‐focused clinical outcomes (Graham et al. 2018; Kuhn and Higgins 2025b; Wilson et al. 2025). Digital tools also supported collaborative goal setting by enabling families and service providers to document, share and revisit goals and to communicate about progress between appointments. In particular, asynchronous communication was considered useful because it allowed goal‐related information to be exchanged without requiring families and professionals to be available at the same time (Khetani et al. 2015; Wilson et al. 2025).

#### 3.4.4 Barriers to Implementation in Practice

Implementation barriers were reported across service settings and framework types: time pressure, high caseloads, administrative workload and travel demand limited service providers' ability to build relationships, coordinate care and sustain family‐centred processes (Carbone et al. 2010; Carroll and Sixsmith 2016; van Maren‐Suir et al. 2018; Wilson et al. 2025). Digital tools could facilitate communication around shared goals, but service providers also expressed concern that incorporating them into already busy practices could create additional demands (Wilson et al. 2025).

Several studies identified tensions between family‐centred approaches and more traditional medical, expert‐led or discipline‐specific models of practice (Callanan et al. 2023; Graham et al. 2018; Jones et al. 2019; Schwellnus et al. 2020). Service providers sometimes experienced holistic family goals as outside their role, difficult to integrate into existing practice or in conflict with expectations for direct intervention and measurable clinical outcomes (Callanan et al. 2023; Jones et al. 2019; Schwellnus et al. 2020).

Information sharing and resource navigation were also challenging aspects of FCP (Carbone et al. 2010; Opoku et al. 2024; Popova et al. 2022). Service providers reported limited awareness of available community resources and difficulty responding to family needs that extended beyond their conventional clinical roles (Carbone et al. 2010; Opoku et al. 2024). Findings from provider measures also indicated that practices related to general information provision were used less consistently than relationship‐building practices (Popova et al. 2022).

#### 3.4.5 Training and Professional Support Needs

Service providers commonly identified a need for training and support in relational, coaching and family‐centred competencies (Callanan et al. 2023; Harbin et al. 2025; Massey et al. 2025; Opoku et al. 2024). They reported that professional education had often focused more on direct child intervention than on caregiver coaching, emotional responsiveness, shared decision‐making and partnership with families (Callanan et al. 2023; Harbin et al. 2025; Opoku et al. 2024).

Training was perceived as more useful when it included mentoring, supervision, feedback, reflective practice or coaching rather than one‐off instruction alone (Callanan et al. 2023; Kuhn and Higgins 2025a; Massey et al. 2025). Service providers also highlighted the need to develop practical coaching skills, including how to facilitate parent‐led interactions, support caregiver confidence and guide families without taking over the intervention process (Callanan et al. 2023; Fabrizi et al. 2024; Harbin et al. 2025; Massey et al. 2025).

#### 3.4.6 Influence of Context and Service Delivery

The implementation of FCP was shaped by service context and delivery mode. Home‐based services were often experienced as supportive of FCP because they allowed professionals to observe children and families within everyday routines and positioned professionals as visitors in the family's environment (Lavigne et al. 2017; van Maren‐Suir et al. 2018). Clinic‐based environments could make family leadership or codelivery more difficult, particularly when services were structured around professional control, equipment or direct intervention routines (Massey et al. 2025).

Remote and teleintervention models introduced both opportunities and challenges for FCP (Harbin et al. 2025; Massey et al. 2025). Remote delivery could increase caregiver involvement because professionals could not physically take over the session, but it also created challenges for modelling, engagement and communication through a screen (Harbin et al. 2025; Massey et al. 2025). Interprofessional collaboration also shaped implementation, as shared training, shared goals and communication across team members were reported to support FCP, while workload and role complexity could make collaboration harder to sustain (Carroll and Sixsmith 2016; Fabrizi et al. 2024; Kuhn and Higgins 2025b).

## 4 Discussion

Across the 21 studies, service providers valued FCP frameworks for supporting collaboration, meaningful goal setting and intervention within everyday contexts. Implementation, however, depended on professional role change, relational competence, training, organisational support and fit with the service setting. This reflects wider evidence that FCP is widely endorsed but inconsistently applied in practice (Kuo et al. 2012; McCarthy and Guerin 2022).

### 4.1 Repositioning Professional Expertise in FCP

A central finding of this review is that FCP frameworks require service providers to reposition their professional expertise. Rather than simply applying a framework or tool, service providers are required to use their expertise in ways that support facilitation, coaching, shared decision‐making and family‐led problem‐solving (Callanan et al. 2023; Graham et al. 2018; Harbin et al. 2025; Schwellnus et al. 2020). This role change was evident across coaching‐based approaches, relational models and collaborative goal‐setting tools, suggesting that FCP requires a shift in how professionals understand therapeutic value, responsibility and expertise (Graham et al. 2018; Jones et al. 2019; Schwellnus et al. 2020; Shie et al. 2025).

This shift is important because FCP should not be interpreted as transferring responsibility from professionals to families. Families may wish to be involved in decision‐making while still needing professional guidance, information and support (King et al. 2017; Kuo et al. 2012; McCarthy and Guerin 2022). FCP therefore depends on shared expertise rather than the replacement of professional expertise by family responsibility (Dunst and Espe‐Sherwindt 2016; King and Chiarello 2014). For paediatric disability services, this means that professionals need to work with families as partners while also providing clear guidance, service navigation and coordinated support (Carbone et al. 2010; MacKean et al. 2005; Popova et al. 2022).

### 4.2 Relational Work and Collaborative Goal Setting as Mechanisms of Practice

The findings indicate that relational and communication competencies are central mechanisms through which family‐centred frameworks are enacted. Service providers' ability to listen, build trust, respond to emotion and understand family priorities shaped whether families could participate meaningfully in decision‐making and intervention planning (Callanan et al. 2023; Carroll and Sixsmith 2016; Graham et al. 2018; Popova et al. 2022). This aligns with broader FCP literature, which identifies respect, partnership, information sharing and empowerment as core components of effective care (Dunst and Espe‐Sherwindt 2016; King et al. 2017). It also aligns with health communication evidence suggesting that clinician–patient communication can influence engagement, trust, shared understanding and emotional support (Street et al. 2009).

Collaborative goal setting was one of the most visible ways in which family‐centred principles were translated into practice. Structured tools and shared planning approaches helped service providers make family priorities more concrete and connect intervention with everyday routines, participation and family life (Jones et al. 2019; Khetani et al. 2015; Lavigne et al. 2017; Rodger et al. 2012; Wilson et al. 2025). These findings are consistent with rehabilitation literature suggesting that collaborative goal setting can improve the relevance of intervention, strengthen caregiver engagement and facilitate meaningful participation through family‐centred partnerships (Crawford et al. 2022; Levack et al. 2006; Watson et al. 2026). In childhood disability services, meaningful goals are particularly important because intervention aims not only to address impairment‐level outcomes but also to support function, participation, family routines and everyday life (Rosenbaum and Gorter 2012).

Relationship‐building and collaborative goal setting also depended on providers' knowledge of available resources, links with relevant services and understanding of whether needs beyond their clinical role should be addressed directly or referred to other services (Carbone et al. 2010; Opoku et al. 2024; Popova et al. 2022).

### 4.3 Frameworks as Enablers, Not Solutions

The five categories reflected the primary function of each approach rather than fixed or mutually exclusive framework types. Some approaches combined several functions: Coaching could support goal setting, while assessment and digital tools could facilitate both planning and communication. Across categories, the frameworks provided practical ways to identify family priorities, develop shared goals and organise collaborative practice, although the available evidence does not establish whether any category is more effective than another (Graham et al. 2018; Jones et al. 2019; Khetani et al. 2015; Lavigne et al. 2017; Wilson et al. 2025).

Frameworks alone did not ensure changes in practice. Some approaches were difficult to incorporate into existing routines and required continued training and supervision (Callanan et al. 2023; Kuhn and Higgins 2025a; Massey et al. 2025). Their use also depended on workforce capacity, organisational support and fit with the local service context (Damschroder et al. 2022; Nilsen 2015).

### 4.4 Implications for Practice and Future Research

Applying an FCP framework requires practical skills as well as knowledge of family‐centred principles. Professional development should address coaching, shared decision‐making, collaborative goal setting, emotional responsiveness and family‐led problem‐solving (Callanan et al. 2023; Harbin et al. 2025; Massey et al. 2025; Opoku et al. 2024). Mentoring, supervision, feedback and reflective practice may be more useful than one‐off training for supporting changes in everyday practice (Callanan et al. 2023; Kuhn and Higgins 2025a; Rush and Shelden 2019).

Training alone, however, cannot address constraints within service delivery. High caseloads, administrative work, travel and limited time restrict relationship‐building and coordination across the included studies (Carbone et al. 2010; Carroll and Sixsmith 2016; Fabrizi et al. 2024; van Maren‐Suir et al. 2018; Wilson et al. 2025). Organisational culture, leadership and resources also shape the implementation of FCP (Damschroder et al. 2022; McCarthy and Guerin 2022). Service managers should therefore consider workload, time for collaborative planning, access to information about community resources and ongoing implementation support.

Future research should examine how training, supervision and organisational support affect the adaptation and sustained use of FCP frameworks across service systems. Research is also needed to determine whether team organisation, funding models and dedicated time for interprofessional collaboration support or restrict their implementation. Studies with larger and more diverse provider samples are needed, particularly outside Western and high‐income settings. Research combining service provider, family and child perspectives could also examine whether frameworks that are feasible for professionals address family and child priorities.

### 4.5 Strengths and Limitations

This review mapped a broad range of FCP frameworks, professional groups and paediatric disability‐related services. The studies, however, varied considerably in design, participants, setting, child population and framework type. Many involved small samples, single teams or one professional discipline. Their findings may therefore reflect local roles and service arrangements rather than the experiences of service providers more broadly.

Formal methodological quality appraisal was not undertaken, consistent with the mapping purpose of a scoping review (Peters et al. 2022; Peters et al. 2021). The recurrence of a theme across studies should therefore be understood as showing its presence in the literature, rather than its prevalence or the strength of the supporting evidence. The review also cannot determine the comparative effectiveness of the frameworks identified.

Screening and data charting were conducted by one reviewer, which may have increased the risk of selection or interpretation bias, although eligibility decisions and findings were discussed regularly with the last author. The review was limited to full‐text empirical journal articles published in English, so evidence in other languages and grey literature may have been missed. As most studies were conducted in Western or high‐income settings, the findings may not transfer directly to other cultural, organisational or healthcare contexts.

## 5 Conclusion

This scoping review found that FCP frameworks provided service providers with practical ways to support collaboration, meaningful goal setting and family‐centred service delivery in paediatric disability‐related services. Frameworks alone, however, are unlikely to produce sustained changes in practice. Their implementation requires professional role development, relational and communication competencies, training, supervision, organisational support and service conditions that enable collaboration with families. Future research should examine how these approaches are adapted and sustained across different service and cultural contexts.

## Author Contributions

**Yanlin Liu:** methodology, writing – original draft, funding acquisition, conceptualization, investigation, data curation, formal analysis, project administration, writing – review and editing, visualization. **Agnes Shiel:** writing – review and editing. **Hazel Killeen:** funding acquisition, writing – review and editing, supervision, conceptualization, methodology, validation.

## Funding

This study was supported by CMNHS Early Career Research grant and China Scholarship Council and Embassy of the People's Republic of China in Ireland (No. 202106370232).

## Ethics Statement

Ethical approval was not required because this scoping review used publicly available published literature and did not involve human participants.

## Consent

Consent was not required because this study was a scoping review of publicly available published literature and did not involve human participants.

## Conflicts of Interests

The authors declare no conflicts of interest.

## Supporting information


**Data S1:** Supporting information.

## Data Availability

Data sharing is not applicable to this article as no new datasets were generated or analysed. The data included in this scoping review are available from the publicly published articles included in the review.
