# Supplementary material for: Service Providers' Perspectives on Family‐Centred Practice Frameworks in Paediatric Disability Services: A Scoping Review
Source: Child Care Health Dev. 2026 Nov 8;52(6):e70376. doi: 10.1111/cch.70376 (PMC13745776; doi:10.1111/cch.70376)
Supplement: Supplementary file 1 [file CCH-52-e70376-s001.docx]

**Supplementary S1 Scoping Reviews (PRISMA-ScR) Checklist**

| **SECTION** | **ITEM** | **PRISMA-ScR CHECKLIST ITEM** | **REPORTED ON PAGE #** |
| --- | --- | --- | --- |
| **TITLE** | | | |
| Title | 1 | Identify the report as a scoping review. | 1 |
| **ABSTRACT** | | | |
| Structured summary | 2 | Provide a structured summary that includes (as applicable): background, objectives, eligibility criteria, sources of evidence, charting methods, results, and conclusions that relate to the review questions and objectives. | 1-2 |
| **INTRODUCTION** | | | |
| Rationale | 3 | Describe the rationale for the review in the context of what is already known. Explain why the review questions/objectives lend themselves to a scoping review approach. | 3-4 |
| Objectives | 4 | Provide an explicit statement of the questions and objectives being addressed with reference to their key elements (e.g., population or participants, concepts, and context) or other relevant key elements used to conceptualize the review questions and/or objectives. | 4 |
| **METHODS** | | | |
| Protocol and registration | 5 | Indicate whether a review protocol exists; state if and where it can be accessed (e.g., a Web address); and if available, provide registration information, including the registration number. | Not registered |
| Eligibility criteria | 6 | Specify characteristics of the sources of evidence used as eligibility criteria (e.g., years considered, language, and publication status), and provide a rationale. | 5-6 |
| Information sources* | 7 | Describe all information sources in the search (e.g., databases with dates of coverage and contact with authors to identify additional sources), as well as the date the most recent search was executed. | 6 |
| Search | 8 | Present the full electronic search strategy for at least 1 database, including any limits used, such that it could be repeated. | 6 |
| Selection of sources of evidence† | 9 | State the process for selecting sources of evidence (i.e., screening and eligibility) included in the scoping review. | 6-7 |
| Data charting process‡ | 10 | Describe the methods of charting data from the included sources of evidence (e.g., calibrated forms or forms that have been tested by the team before their use, and whether data charting was done independently or in duplicate) and any processes for obtaining and confirming data from investigators. | 7 |
| Data items | 11 | List and define all variables for which data were sought and any assumptions and simplifications made. | 7-8 |
| Critical appraisal of individual sources of evidence§ | 12 | If done, provide a rationale for conducting a critical appraisal of included sources of evidence; describe the methods used and how this information was used in any data synthesis (if appropriate). | Not conducted |
| Synthesis of results | 13 | Describe the methods of handling and summarizing the data that were charted. | 8 |
| **RESULTS** | | | |
| Selection of sources of evidence | 14 | Give numbers of sources of evidence screened, assessed for eligibility, and included in the review, with reasons for exclusions at each stage, ideally using a flow diagram. | 8-9 |
| Characteristics of sources of evidence | 15 | For each source of evidence, present characteristics for which data were charted and provide the citations. | 9-15 |
| Critical appraisal within sources of evidence | 16 | If done, present data on critical appraisal of included sources of evidence (see item 12). | Not conducted |
| Results of individual sources of evidence | 17 | For each included source of evidence, present the relevant data that were charted that relate to the review questions and objectives. | 16-19 |
| Synthesis of results | 18 | Summarize and/or present the charting results as they relate to the review questions and objectives. | 20-28 |
| **DISCUSSION** | | | |
| Summary of evidence | 19 | Summarize the main results (including an overview of concepts, themes, and types of evidence available), link to the review questions and objectives, and consider the relevance to key groups. | 28-32 |
| Limitations | 20 | Discuss the limitations of the scoping review process. | 32-33 |
| Conclusions | 21 | Provide a general interpretation of the results with respect to the review questions and objectives, as well as potential implications and/or next steps. | 33 |
| **FUNDING** | | | |
| Funding | 22 | Describe sources of funding for the included sources of evidence, as well as sources of funding for the scoping review. Describe the role of the funders of the scoping review. | 33 |

JBI = Joanna Briggs Institute; PRISMA-ScR = Preferred Reporting Items for Systematic reviews and Meta-Analyses extension for Scoping Reviews.

* Where *sources of evidence* (see second footnote) are compiled from, such as bibliographic databases, social media platforms, and Web sites.

† A more inclusive/heterogeneous term used to account for the different types of evidence or data sources (e.g., quantitative and/or qualitative research, expert opinion, and policy documents) that may be eligible in a scoping review as opposed to only studies. This is not to be confused with *information sources* (see first footnote).

‡ The frameworks by Arksey and O’Malley (6) and Levac and colleagues (7) and the JBI guidance (4, 5) refer to the process of data extraction in a scoping review as data charting*.*

§ The process of systematically examining research evidence to assess its validity, results, and relevance before using it to inform a decision. This term is used for items 12 and 19 instead of "risk of bias" (which is more applicable to systematic reviews of interventions) to include and acknowledge the various sources of evidence that may be used in a scoping review (e.g., quantitative and/or qualitative research, expert opinion, and policy document).

*From:* Tricco AC, Lillie E, Zarin W, O'Brien KK, Colquhoun H, Levac D, et al. PRISMA Extension for Scoping Reviews (PRISMAScR): Checklist and Explanation. Ann Intern Med. 2018;169:467–473. [doi: 10.7326/M18-0850](http://annals.org/aim/fullarticle/2700389/prisma-extension-scoping-reviews-prisma-scr-checklist-explanation).

**Supplementary S2 Search strategy for the scoping review of service providers’ perspectives on family-centred practice frameworks**

## Table 1. Search concepts and keywords

| **Search line** | **Search terms** |
| --- | --- |
| Population (Service providers) | (service provider* OR professional* OR health* personnel OR occupational therapist* OR speech and language therapist* OR physiotherapist* OR psychologist* OR social worker* OR clinician* OR doctor* OR therapist* OR manager* OR researcher*) |
| Concept (Family-centred practice or frameworks) | ("family centred care" OR "family-centered care" OR "family centred practice" OR "family-centered practice" OR "family centred framework*" OR "family-centered framework*" OR "family integrated care") |
| Population served (Children) | (child* OR infant* OR toddler* OR adolescent* OR youth* OR teenager* OR pediatric* OR paediatric*) |
| Population served (Disability) | (disabilit* OR disable* OR impair* OR handicap* OR "special needs" OR developmental* OR "developmental delay" OR "cerebral palsy" OR autism OR ASD OR asperger* OR blind* OR "low vision" OR "sight loss" OR deaf* OR "hearing loss") |
| Outcome (Perspectives) | (perspective* OR perception* OR attitude* OR experience* OR view* OR understanding*) |

The search strategy comprised five concept groups: service providers, family-centred practice, children and young people, disability-related conditions, and perspectives and experiences. Within each concept group, keywords and synonyms were combined using the Boolean operator OR. The five concept groups were then combined using AND. Truncation (*) and phrase searching were used where appropriate. Search terms were adapted to the indexing requirements and search syntax of each database..

## Table 2. Database search summary

| **Database** | **Date searched** | **Search fields** | **Results identified** |
| --- | --- | --- | --- |
| CINAHL Complete | 29-Jan-26 | Subject headings, title, abstract and keywords | 834 |
| APA PsycINFO (via EBSCOhost) | 29-Jan-26 | Subject headings, title, abstract and keywords | 413 |
| SocINDEX with Full Text (via EBSCOhost) | 29-Jan-26 | Subject headings, title, abstract and keywords | 190 |
| Academic Search Complete (via EBSCOhost) | 29-Jan-26 | Title, abstract and keywords | 1055 |
| Scopus | 29-Jan-26 | Article title, abstract and keywords | 189 |
| MEDLINE (via PubMed) | 29-Jan-26 | MeSH terms and title/abstract fields | 459 |

Total records identified before deduplication: 3,140

## Database-specific adaptations

The five search concept groups remained consistent across all databases. Minor modifications were made to accommodate differences in indexing systems, controlled vocabulary, subject headings, field codes and search syntax.

CINAHL Complete, APA PsycINFO, Academic Search Complete and SocINDEX with Full Text were searched through the EBSCOhost platform. Database-specific subject headings were used where available, together with title, abstract and keyword searching. Scopus was searched using article title, abstract and keyword fields. MEDLINE via PubMed incorporated relevant Medical Subject Headings (MeSH) together with title and abstract terms.

Where supported by the database, filters for academic journal records and abstract availability were applied. No date restrictions were applied. English-language publication was applied as the review-level eligibility criterion during study selection.
